# Multichannel Genomic Recording of Biological Information with ENGRAM

**DOI:** 10.1038/s41596-025-01322-w

**Published:** 2026-02-11

**Authors:** Jenny F. Nathans, Troy A. McDiarmid, Wei Chen, Jay Shendure

**Affiliations:** 1. Department of Genome Sciences, University of Washington, Seattle, WA, USA; 2. Medical Scientist Training Program, University of Washington, Seattle, WA USA; 3. Seattle Hub for Synthetic Biology, Seattle, WA, USA; 4. Department of Biochemistry, University of Washington, Seattle, WA, USA; 5. Institute for Protein Design, University of Washington, Seattle, WA, USA; 6. Brotman Baty Institute for Precision Medicine, Seattle, WA, USA; 7. Howard Hughes Medical Institute, Seattle, WA, USA; 8. Allen Discovery Center for Cell Lineage Tracing, Seattle, WA, USA

## Abstract

Molecular recording is an emerging paradigm for measuring biology over time. ENGRAM (**EN**hancer-mediated **G**enomic **R**ecording of **A**ctivity in **M**ultiplex) is a recently described synthetic biology circuit architecture that converts the transient activity of *cis*-regulatory elements (CREs) into stable genomic records that can be retrospectively recovered via DNA sequencing. Here we provide a step-by-step protocol for conducting ENGRAM experiments and analyzing the resulting data. We also describe key design considerations for ENGRAM recorders, summarize the strengths and limitations of ENGRAM, and highlight applications including multiplex signal recording and high-throughput CRE screening. For users with basic skills in molecular biology, mammalian cell culture, and DNA sequencing analysis, ENGRAM experiments can typically be completed within 5–6 weeks. In contrast with other systems for DNA-based recording in mammalian systems, ENGRAM relies on prime editing-mediated insertions to record the activity of a given CRE, such that it is inherently multiplexable, *e.g*. 4-bp insertions can represent the activities of up to 256 distinct CREs. A further contrast lies with ENGRAM’s compatibility with DNA Typewriter, which facilitates the capture of signal order.

## Introduction

The emerging paradigm of molecular recording seeks to monitor cellular activities and stably preserve this information within cells for retrospective recovery^1,2^. Genomic DNA is an arguably ideal medium for both encoding the machinery of molecular recorders (*i.e*. leveraging the transcriptional and translational machinery of the cell to build recorders from within each cell with atomic precision), as well as for serving as the substrate for information storage (*i.e*. leveraging the stability, biocompatibility and digital nature of DNA, together with high-throughput DNA sequencing technologies for the recovery of records^3^). A DNA-based molecular recorder typically comprises two key components: (i) a DNA writer: an enzyme that writes records by altering a target DNA sequence in a biologically conditional manner; and (ii) a DNA Tape: an endogenous or engineered region of the genome that serves as the recording substrate for the DNA writer.

The field of DNA-based molecular recording has recently gained momentum due to progress in genome engineering, especially CRISPR-related technologies^4–10^. Early recorders used recombinases to invert or excise specific DNA sequences^11–13^. More recent CRISPR nuclease-based recorders leverage cells’ DNA repair mechanisms to generate diverse mutations at a DNA Tape after Cas9-mediated double-strand break repair^4,5^. Alternatively, base editing allows for precise point mutations within a defined sequence window^6,7,14–16^. These CRISPR-based methods are arguably more effective than recombinase-based methods for stochastically creating sequence diversity, which is particularly useful for applications such as cell lineage tracing.

To move beyond recording cell lineage, biologically conditional activation of either recombinase- or CRISPR-based recorders can be achieved using tissue-specific or signal-responsive CREs driving the expression of editing enzymes^6,7,14,16^. However, most such approaches are limited with respect to multiplexing, particularly if a different enzyme and/or DNA Tape is required for each signal. It is challenging to imagine how one would concurrently record and recover dozens to hundreds of signals in the same system with such constraints.

**Figure 1.**
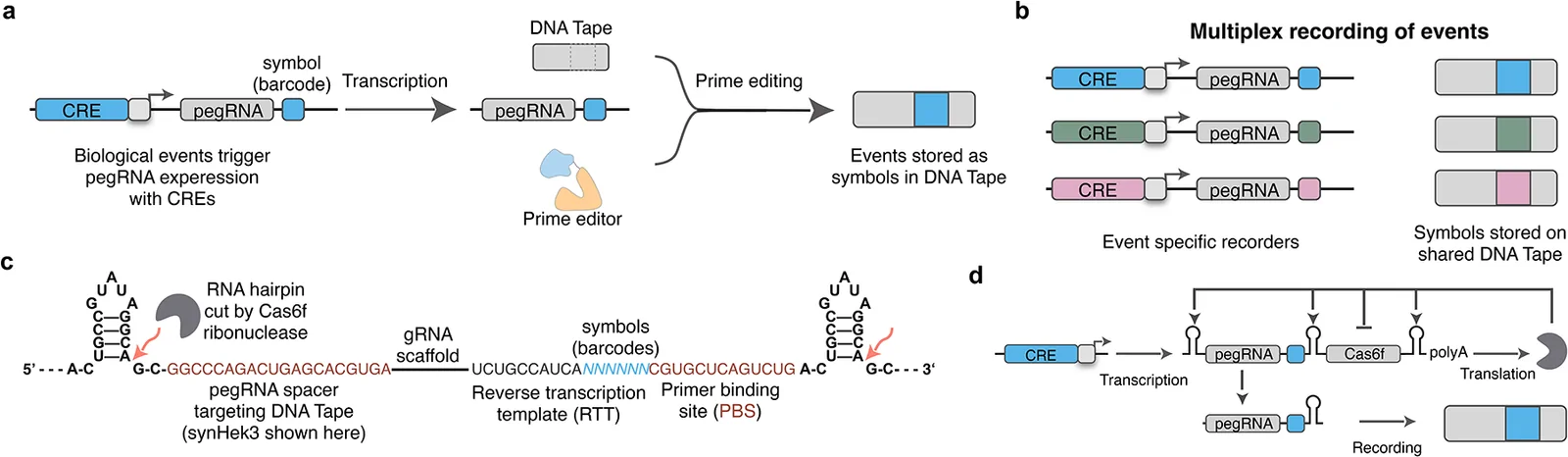
Architecture of ENGRAM. **a**. Schematic of ENGRAM. *Cis*-regulatory element (CRE) activity drives the expression of a pegRNA encoding an insertional barcode (*i.e*. symbol) unique to the CRE. **b**. Multiplex or multichannel recording is achieved by constructing and concurrently introducing signal- or state-specific recorders, each encoding a unique symbol. The probability of a given symbol being written to the DNA Tape is a function of the expression level of its encoding pegRNA, which is in turn a function of CRE activity. **c**. The Pol-2-driven pegRNA is flanked by two 17 bp *cas6f* (*csy4*) hairpins and can be released by the endonuclease Cas6f (Csy4). All pegRNAs share the same spacer sequence (targeting a shared DNA Tape) and vary only in the encoded insertion (symbol). **d**. A feed-forward loop and an AND-gate circuit in 5’-ENGRAM that potentially explains the improved signal-to-noise that we observe relative to alternative architectures. Cas6f cleavages its own mRNA, resulting in the release of pegRNA and inhibition of further Cas6f production. Arrows: activation. Bar: inhibition. This figure is partially adapted from various panels of Figure 1 of the ENGRAM publication^17^.

To record signal-specific events in cells in a manner that is compatible with extensive multiplexing, we and colleagues recently developed ENGRAM^17^—a prime editing-based system that converts the activity of *cis*-regulatory elements into the production of pegRNAs encoding insertional DNA barcodes (Figure 1a,b). These barcodes, termed symbols, are then inserted into a DNA Tape by the prime editor. Each ENGRAM unit comprises an activity-specific CRE driving the production of a pegRNA encoding an CRE-specific symbol (Figure 1a). The number of simultaneously recordable signals scales exponentially with symbol length (*i.e*. an n-mer insertion can potentially encode the activities of 4^n^ distinct CREs). ENGRAM can also be combined with DNA Typewriter, a complementary technology that we concurrently developed for sequential genome editing^18^. Together, DNA Typewriter and ENGRAM enable genomic recording of the timing and order of multiple CRE activities^17^.

This protocol outlines how to design an ENGRAM experiment, including key steps for cloning ENGRAM recorders and for creating recording-compatible cell lines. In addition, we describe the retrieval of recorded information from cells, which should be straightforward for those with experience in high-throughput sequencing of PCR amplicons. We showcase ENGRAM’s potential by highlighting its application to: (i) recording the activity and orders of two canonical pathways, Wnt and NFκB; and (ii) profiling the activity of hundreds of enhancers in cells. For experiments focused on recording the order of signals, please refer to our separate DNA Typewriter protocol^19^.

## Architecture of ENGRAM

In our view, a robust, inherently multi-channel DNA-based molecular recording system has two key requirements. First, a unique symbol must be assignable to each signal. An alternative approach uses different enzymes, each conditional on a different signal, to edit distinct target sequences, but the number of enzymes quickly becomes limiting. The CRISPR/Cas9 system simplifies this by allowing the design of unique gRNA-target pairs, with each pair representing a distinct signal. However, recording different signals to different locations makes it more challenging to recover information, and also to make inferences about how these signals relate to one another in terms of order and strength. In contrast, prime editing enables one to write a large number of unique symbols, in the form of insertional edits, to a shared location(s). This greatly simplifies both the writing and reading of DNA-based records.

Second, the writing of a given symbol must be conditional on the activity of its associated biological signal. Guide RNAs, including prime editing guide RNAs (pegRNAs), are conventionally expressed in mammalian systems under the control of constitutive Pol-3 promoters (*e.g*. the U6 promoter), which lack inducibility in response to biological states or signals. While Pol-2 promoters allow for conditional gene expression, the resulting transcripts undergo modifications such as 5’ capping and 3’ polyadenylation, which may impede prime editing activity. Strategies to release functional pegRNA from Pol-2 transcripts include using self-cleaving ribozymes^20^, tRNA processing machineries^21^, and ribonucleases^22^. Although these or other alternatives are likely possible, for ENGRAM’s proof-of-concept, we used the ribonuclease Cas6f (also known as Csy4), which specifically recognizes and cleaves a 17-bp *cas6f* hairpin structure (Figure 1c). Constitutive expression of Cas6f, independent of the pegRNA, enabled recording but also resulted in high background activity. To address this issue, we designed co-expressing architectures in which the pegRNA is embedded into the untranslated regions (UTRs) of the transcript of Cas6f (Figure 1d). Given that Cas6f acts as a single-turnover enzyme^23^, this design creates a coherent feedforward loop with an AND gate, effectively reducing background activity while maintaining strong signal responsiveness (Figure 1d). Although we continue to explore additional ENGRAM architectures, we currently recommend Cas6f/*cas6f* and 5’-ENGRAM (pegRNA embedded in the 5’ UTR) for its high signal-to-noise and simplicity in cloning.

## Applications of ENGRAM

**Figure 2.**
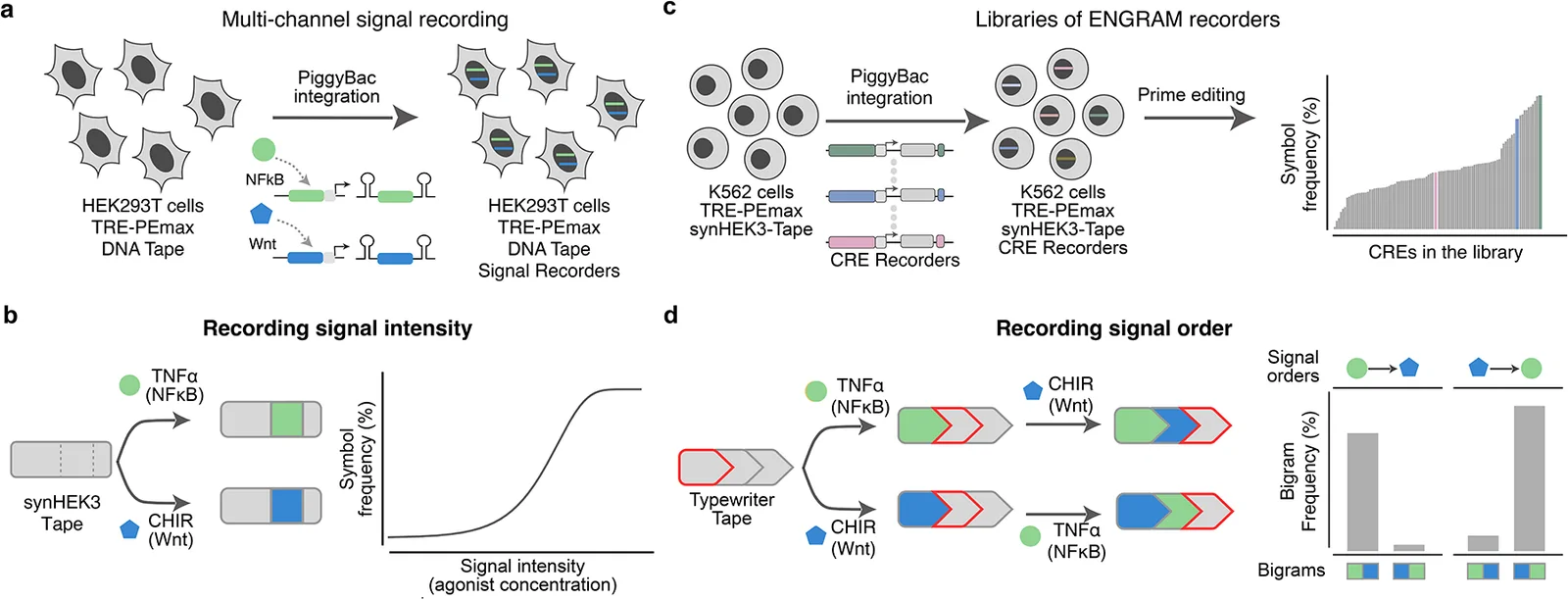
Applications of ENGRAM. **a**. Illustrative example of components for multi-channel signal recording with ENGRAM, including a cell line (*e.g*. HEK293T or K562) or organism bearing an inducible writer (*e.g*. Dox-inducible PEmax) and one or many copies of DNA Tape (*e.g*. synHEK3 or DNA Typewriter). We generally introduce these components through PiggyBac-based random integration, although targeted integration is possible. **b**. Recording signal intensity. In dose-response experiments conducted over a fixed time frame, we have found that the relative frequencies at which a signal-specific symbol in DNA Tape are observed is a nonlinear function of the concentration of the corresponding agonist. **c**. Library screening of CRE activities. A library of CREs can be associated with specific pegRNA-encoded insertions during cloning. The resulting library of ENGRAM recorders can then be integrated to target cells bearing an inducible writer (*e.g*. Dox-inducible PEmax) and one or many copies of DNA Tape (*e.g*. synHEK3 or DNA Typewriter). The relative activities of the many CREs can be quantified by sequencing the abundance of their corresponding symbols in DNA Tape, *i.e*. a massively parallel recorder assay rather than a massively parallel reporter assay. **d**. As proof-of-concept of recording the order of signals, cells bearing pre-integrated inducible writer, DNA Typewriter Tape, and two signal-specific ENGRAM recorders, were treated with the corresponding agonists in various patterns, the simplest pattern being to simply switch from one agonist to the other, or vice versa. We found that these and other ordering patterns were readily distinguishable based on the bigrams of symbols observed in an ensemble of DNA Typewriter Tapes. This figure is partially adapted from various panels of Figures 2–4 of the ENGRAM publication^17^.

Thus far, we and colleagues have applied ENGRAM to: (i) recording the activity and orders of signaling pathways in mammalian cells; (ii) concurrently recording dozens to hundreds of CREs’ activities. We have shown that signaling transductions that regulate transcription, such as exposure to a Wnt-pathway agonist, can be quantitatively recorded with ENGRAM (Figure 2a–b). To record signaling events or cell states, one only has to pair each signal- or state-specific CRE with a pegRNA-encoded insertional symbol. This is readily achievable in a library format, as hundreds of ENGRAM recorders, each bearing a different CRE, can be concurrently cloned in *cis* with CRE-specific pegRNAs and used in a single experiment (Figure 2c). The number of CREs whose activities can be distinguished within the context of a single system or experiment increases exponentially with the length of the inserted symbols. For example, a 4-bp symbol allows for unique assignment of up to 256 (4^4^) CREs, while a 5-bp symbol allows for unique assignment of up to 1024 (4^5^) CREs. The presence and magnitude of a given signal can be inferred from the abundance of the corresponding symbol in DNA Tape (Figure 2b–c). Furthermore, combining ENGRAM with DNA Typewriter^18^ enables one to record the order of CRE activities by structuring the DNA Tape in a manner that is sequentially editable (Figure 2d). To demonstrate this concept, we showed that various patterns of two signals are distinguishable from one another by the patterns of ‘bigrams’ that appear in DNA Typewriter Tape^17^.

In our proof-of-concept report of ENGRAM^17^, we demonstrated its potential to reliably record several orthogonal signaling pathways (*e.g*. Tet-On, NF-kB, Wnt) and its compatibility with various mammalian *in vitro* models, including HEK293Ts, K562s, and a mouse ESC (mESC)-derived gastruloids. Prime editing itself remains a relatively recent development in genome engineering, and we anticipate that ENGRAM will be readily adaptable to other models in which prime editing has been demonstrated, including other cancer cell lines, human organoids, mice^24–27^ and possibly other organisms. In our hands, we have had success with ENGRAM in the cell lines described above, as well as mESCs, mESC-derived multicellular gastruloids and embryoid bodies. We and others are actively pursuing ENGRAM recording in mice *in vivo* and other multicellular systems *in vitro*, including hiPSC-derived organoids. Potential challenges to such adaptations include that Cas6f toxicity may be limiting in some systems (discussed further below), as well as delivery and/or silencing of recording machinery over time. If encountered, such challenges can in principle be overcome with alternative gRNA release mechanisms^20,28^, improved and/or multipronged delivery strategies^29^, or consolidating all recording machinery in optimized configurations at silencing-resistant safe-harbor loci^30^. We also anticipate that the basic architecture can be adapted to myriad CREs that are either known to be, or can be engineered to be, biologically conditional. These include other signaling pathways, cell types, cell states, and possibly even signals that do not endogenously result in transcriptional changes (*e.g*. synNotch-triggered Pol-2 transcription of ENGRAM-released pegRNAs to record specific cell-cell or protein-protein interactions of interest^31,32^).

## Comparison with other methods

### Signal Recording

The rapidly evolving toolkit of genetic engineering methods has enabled the permanent recording of transcriptional activity into genomic loci to label a population of cells over time. Although a substantial base of research has developed molecular recording tools in bacteria^8,9,33^, here we focus exclusively on molecular recording in mammalian systems.

Molecular recording technologies that rely on the heritable gain or loss of a fluorescent marker in response to a signal-driven genome editor, *e.g*. site-specific recombinases, are limited in ability to multiplex by the number of spectrally distinct fluorescent channels and by the lack of orthogonal enzymes that target distinct sequences^11,34,35^. Techniques utilizing DNA base editors have demonstrated event-triggered molecular recording either through direct sequencing of the edited locus or successive rounds of FISH probe hybridization of the edited locus or loci^6,7,15^. These methods may additionally provide information about the sample’s spatial architecture if they rely on an optical readout, such as FISH.

Unlike recorders based on DSBs, ENGRAM utilizes a nickase and exclusively relies on Pol-2-driven pegRNAs for recording, and so can be used to simultaneously and sequentially record many different events written as unique insertions to a shared DNA Tape. Base editors can also utilize Pol-2 transcribed guides, but a given recording site for base editing can only exist in one of two states, such that each signal of interest would require its own set of recording sites for multiplex signal recording. The multiplexing capacity of ENGRAM is primarily limited by prime editing efficiency, as dividing prime editing capacity between many different elements may make it more challenging to distinguish signal from noise. However, prime editing efficiency continues to improve with advances in the field, including optimizing the prime editor protein, modulating endogenous cellular factors that inhibit or activate prime editing, and stabilizing the pegRNA^36–39^.

Other tools to record cellular events in mammalian cells include circuits to record high and low miRNA expression through miRNA mediated cleavage of a gene or endoribonuclease from an mRNA backbone. This results in the selective destabilization or stabilization of desired transcripts^40^. Intriguingly, this was utilized not only to report on miRNA levels, but also to drive cell state transitions through the selective stabilization of RUNX1, a transcription factor that guides pluripotent cells towards a hematopoietic lineage. As ENGRAM similarly relies on endoribonucleases as tools to release effectors from mRNA structural elements, one can imagine analogous modifications that enable ENGRAM to encode signal-activated programs (*e.g*. a CRE drives the production not only of a pegRNA that drives a recording event, but also of a pegRNA or gRNA that mediates a cell fate-modulating genetic perturbation).

### Library Screening

Many of the technologies described above have been applied to, at most, the recording of 2–3 concurrent signals, enhancer activities, or cellular events rather than high complexity libraries. In contrast, massively parallel reporter assays (MPRAs) are commonly used to measure the activity of thousands of different CREs in multiplex^41^. In the MPRA framework, CRE activity results in the transcription of RNA molecules linked to CRE-specific barcodes. Recent advances in the field have developed MPRA constructs capable of distinguishing CRE strength on a single-cell level by utilizing a built-in control for construct abundance, a U6-driven circular barcode that is highly captured by 10× 3’ scRNA sequencing and used to normalize a Pol-2 enhancer-driven barcode on the same construct^42^. In the MPRA paradigm, the measurements of enhancer strength are transiently reported rather than permanently recorded, which may make it more challenging to interpret cellular history.

In contrast, ENGRAM records the activity of CRE and signal-response elements permanently either to endogenous genomic loci or integrated synthetic DNA Tape (*e.g*. synHEK3 Tape or DNA Typewriter Tape). We previously demonstrated that ENGRAM is capable of recording the activity of at least hundreds of endogenous CREs and synthetic transcriptional factor (TF) binding CREs (synCREs) in mammalian cells, including in a model of early mammalian development. The CRE activities recorded on DNA Tape showed a strong correlation with those measured by RNA-seq. We further demonstrated that cells containing multiple synthetic DNA Tape integrations exhibit increased recording capacity, thereby reducing the number of cells needed to accurately recover CRE activities. Moreover, because ENGRAM permanently records activity in the DNA Tape, it can capture transient CRE activities that may be missed by MPRA, which only reflects activity at the time of measurement. Taken together, ENGRAM enables new possibilities for how we study the dynamics of signaling and CRE activity during complex biological processes such as embryonic development and cancer metastasis.

### Recording the order of signaling events

ENGRAM pegRNAs can be engineered to target synthetic DNA Tapes that are sequentially editable, as in both the DNA Typewriter and peCHYRON systems^18,43^. In DNA Typewriter, a synthetic array of target sites is integrated into the cellular genome. Before any of these targets are edited, all but the first are truncated by 3-bp at their 5’ end - these are missing a short 3-bp sequence called the “key”, which is necessary for a successful prime editing event to occur as it completes the target sequence. When an insertion is edited into this synthetic array, the insertion is placed at the 5’ end of the next site of the synthetic array, and this insertion includes the key. The insertion of the key completes the target site of the next unit in this synthetic array, thus enabling its editing by prime editing machinery. We have shown that CRE driven pegRNAs from the ENGRAM system can target this DNA Typewriter synthetic target array (*e.g*. of a succession of monomers whose compatibility with editing is gated on the monomer 5’ to it being first edited), thus enabling us to record sequentially resolved signaling and CRE activities. The peCHYRON system differs from DNA Typewriter in that it sequentially inserts an event specific sequence alongside the entire next DNA Tape to be edited, rather than *a priori* integrating the DNA Tape targets - this allows editing to continue indefinitely. While not yet demonstrated, we see no technical reason why ENGRAM-derived pegRNAs could not be used to enable Pol-2-driven sequential recording with peCHYRON DNA Tapes as well.

In addition to nucleic-acid based mediums of sequentially-resolved molecular recording, protein fibers have been used to record cellular events as well^44,45^. In these systems, both engineered and computationally designed self-assembling protein monomers containing epitope tags are expressed either constitutively (*e.g*., through the UbC promoter) or in response to a cellular event by utilizing endogenous gene promoters or signal response elements (*e.g. cFos, Egr1*, and *Arc* promoters, or the CREB promoter). These monomers polymerize in cells into flexible, optically visible fibers that may be visualized via immunofluorescent imaging. Recent advances have resulted in the development of modular monomers capable of accommodating diverse epitopes that may all contribute to the same fiber, thus enabling multiplexed, sequentially resolved recording of cellular events over the course of days to weeks^46^. This recorded information inherently includes spatial context, as it is read optically from the cells. Compared with these methods, ENGRAM/DNA Typewriter offers several advantages. Firstly, the DNA Tape provides a relatively compact storage medium in comparison to protein fibers that are large enough to be optically viewed. Secondly, all information stored to DNA Tapes across many cells with the ENGRAM/DNA Typewriter system may be read at once in the same sequencing run; in order to read-out multiplexed signals that have recorded to the same protein fiber, multiple rounds of immunofluorescence must be performed, which becomes more time-intensive as the number of unique signals increases. Thirdly, ENGRAM/DNA Typewriter can be readily scaled to sample millions of cells, whereas an optical read-out is limited in field of view. Finally, we anticipate that ENGRAM/DNA Typewriter records can be adapted to spatial sequencing read-outs as spatial sequencing tools proliferate.

## Limitations of ENGRAM

Limitations of ENGRAM stem from current limitations in the field of genome and cell engineering, DNA synthesis, and synthetic circuit design.

### Prime editing (PE) efficiency:

The rate of recording with ENGRAM is intrinsically linked to the PE efficiency in the cellular context/s of interest. Low PE efficiency can make it difficult to reliably detect transcriptional events, or discern signals from noise. Low PE efficiency can occur in some cell types due to altered DNA repair mechanisms^36^, nucleotide availability^47^, etc. and can require optimization. Strategies to achieve high efficiency PE in many diverse contexts have been reported^48^ and are advancing rapidly. ENGRAM records reflect both signal intensity and duration, so precise determination of signal duration and intensity requires the use of molecular timestamps (i.e. ENGRAM records with known absolute timepoints of induction to reconstruct timing of other records). While we do not know exactly how fast a given ENGRAM record can be installed after transcription is triggered, our current estimate is on the order of a few hours, based on the general kinetics of genome editing in mammalian cells, and the fact we could resolve the order of two signals delivered 24h apart^17^. As such, ENGRAM can be used to record many signals over long time periods, but it is unlikely to be well suited to decoding fast signal kinetics (*e.g*. seconds, minutes).”

### Symbol length & number:

The number of signals that can be simultaneously recorded, and the accuracy of recording for a given signal, is linked to the length and number of barcodes that can be paired to each signal/CRE, delivered to the cells, and written to the genome. Too few or too short barcodes for a given library size can lead to inaccurate measurements or difficulty in demultiplexing records from independent signals (from errors in barcode replication, insertion, or read out). The longer the insertions that can be reliably achieved in a system, the more signals/CREs and barcodes per signal/CRE (which improves measurement accuracy) can be simultaneously measured. Pooled ENGRAM screens of hundreds of signals/CREs have already been achieved in multiple systems^17^, and improved PE efficiency along with more and longer barcodes per signal/CRE should enable recording screens at the scale of MPRAs (hundreds of thousands of sequences)^49^ in the near future.

### Construct stability & design:

ENGRAM, like many synthetic circuits, uses well-characterized parts (*e.g*. gRNA scaffolds, Cas6f/*cas6f* hairpins, minimal promoters, etc.) repeatedly in a modular fashion. This design enables accurate comparison of signal/CRE activity but also introduces repetition into construct designs. Repetitive constructs can pose challenges to DNA synthesis, cloning, and long term stability in cells^50–53^. Highly repetitive arrays of multiple ENGRAM constructs could lead to assembly failures, deletion or silencing of certain components. Problematic circuit designs where individual ENGRAM cassettes are not properly distanced or insulated from one another could also cause crosstalk between enhancers^54^ in neighbouring ENGRAM units. Diversified parts for genome engineering and molecular recording are being developed (*e.g*. diversified gRNA scaffolds capable of PE)^55^, as are improved methods for large-scale DNA assembly^56,57^, and modular cloning strategies to test diversified parts^58^ that are all simplifying recorder design and assembly and in use in our laboratory.

### Bioorthogonality:

Cas6f may be cytotoxic in certain contexts^22^, though this is mitigated in ENGRAM by the transient and highly destabilized nature of the ENGRAM transcript. PE, and consequently ENGRAM, does not rely on DNA DSBs the way that recorders based on Cas nucleases such as GESTALT do^59^, and as such may be less disruptive. Nonetheless, PE, like all genome editors and exogenous proteins, will inevitably have effects on cellular physiology^60^ that should be monitored for.

### Silencing of recording machinery:

ENGRAM requires at least 2 recording components, the prime editor and ENGRAM cassette, to maintain high transcription capability throughout the intended recording time period. ENGRAM circuits that use synthetic TFs to drive recording require additional components with stable expression. Silencing^30^ of one or more of the recording components over time can lead to failure to record signals/events that are active at later time points and/or more differentiated cell states. Improved silencing resistant constructs that enable delivery and stable expression of large payloads^61^ along with improved assembly strategies (discussed above) enable consolidating recording machinery to single constructs that can all be integrated at safe harbor loci which are less prone to silencing and simplify breeding/distribution (*e.g*. for disease modeling).

### Overview of procedures

Here we provide a detailed protocol for performing ENGRAM experiments. In particular, this protocol provides detailed instructions for creating ENGRAM constructs and generating suitable cell lines for recording cellular events. The process involves: Cloning UMI-barcoded DNA Tapes (**Steps 1 −8**); Generating a cell line with inducible PEmax expression and integrated DNA Tapes (**Steps 9–21**); Cloning ENGRAM constructs with specific CRE and symbol pairings (**Steps 22–23**); Generating cells with all recording components (**Steps 23–29**); Recording signal intensities (**Steps 30–34**) and orders (**Steps 35–40**); Recording and screening CRE activities (**Steps 35–40**). Recurrently used procedures such as Golden Gate Assembly and retrieval of recorded information from cells are summarized into Box 1 and Box 2, respectively.

### ENGRAM experimental design

Several key considerations need to be addressed when designing ENGRAM experiments:

1. **Choice of DNA writer:** The efficiency of prime editing is critical for successful molecular recording with ENGRAM. At present, PEmax is the recommended default due to its high efficiency.
2. **ENGRAM Architecture:** Both 5’-ENGRAM and 3’-FT-ENGRAM exhibit strong response to stimulation. 3’-FT-ENGRAM exhibits lower background noise and slightly higher activation, potentially improving the dynamic range of recording. However, for ease of cloning and minimized CRE-BC distance, we generally recommend 5’-ENGRAM. Throughout this protocol and in our ongoing work, we are using 5’-ENGRAM. We also provide example cloning files for 3’-FT ENGRAM as reference.
3. **Choice of cell line**: While transient transfection of ENGRAM recorders and prime editor plasmids can be used for signal recording and library screening, we recommend generating a monoclonal cell line with multiple copies of DNA Tapes and stable expression of PEmax for more robust and consistent results. ENGRAM has been successfully implemented in HEK293T, K562, and mouse embryonic stem cells (mESCs). This protocol will use HEK293T cells as an example for generating a stable cell line suitable for recording experiments. Specific culturing conditions for other cell lines are beyond the scope of this protocol.
4. **Recording Order of Events:** Determine if recording the temporal order of events is necessary. ENGRAM can be integrated with DNA typewriter technology to capture the sequence of signals. While recording to the DNA Typewriter Tape is less efficient than targeting the synHEK3 Tape (or endogenous HEK3 locus), we have developed a cloning strategy that allows for easy exchange of DNA Tape and pegRNAs between the synHEK3 Tape and Typewriter Tape.
5. **Choice of CREs and Symbols:** Carefully select the cis-regulatory elements (CREs) for signal recording and screening, as well as the symbols to be associated with these CREs. Certain CREs exhibit low baseline activity and high induced activity (which are better suited to recording the presence or absence of an event), while others have high baseline activity and higher stimulated activity (which can be better for continuous recording of signaling dynamics). CREs can also vary in size by orders of magnitude (typically ~100–1000bp). Longer endogenous sequences defined by chromatin accessibility may better reflect endogenous CRE activity, while shorter synthetic CREs may yield more specific, tailorable responsiveness.
6. **Barcode Selection:** The choice of barcodes should be based on the complexity of the library. A Hamming distance of 1 is sufficient to distinguish different barcodes using this protocol. We have observed variations in recording efficiency among different barcodes and have curated lists of 5-bp and 8-bp insertion efficiencies. For recording a limited number of signaling pathways, 5-bp barcodes should suffice. For screening thousands of CREs, we recommend using 8-bp barcodes to mitigate barcode collapse.
7. **Control selection and interpretation:** If the pool of CREs has less than ~5000 elements, we recommend two or more barcodes per CRE to help control for insertion differences between barcodes (*i.e*., the impact of such differences are mitigated by the calculation of edit scores, but consistent results between edit scores derived from independent barcodes will add confidence). In general, longer and more independent insertion barcodes per CRE will improve recording accuracy, but potentially at the cost of reduced recording efficiency. We have recorded libraries of hundreds of CREs with 5N barcodes and libraries of thousands of CREs with 8N barcodes. We also strongly recommend spiking in “empty” ENGRAM recorders with a minimal or no promoter driving one or several barcodes as a negative control to determine the noise floor *(e.g*., with no minP, with minP alone, and/or with yeast or bacterial elements that should not be active in mammalian cells). Active CREs should drive recording levels higher than those of these negative controls^17^. The assessment of whether a given CRE is active should be performed with appropriate statistics that take into account independent barcodes, technical replicates, etc., but as a subjective ballpark, we look for activity at least 2-fold greater than minP negative controls. Where needed, positive controls for ENGRAM can take the form of recorders driven by constitutive promoters (*e.g*. CMV) or a dox-inducible CRE^17^. However, a caution is that if positive controls are too active, they can dominate the set of recorded events and potentially compromise quantification of signal recording.”

## Materials

### Biological materials

- NEB Stable competent cells (NEB, cat. no. C3040H).
- NEB 10-beta electrocompetent cells (NEB, cat. no. C3020K).
- HEK293T (ATCC, cat. no. CRL-3216; RRID: CVCL_0063) or desired target cell line.
- K562 (ATCC, cat. no. CCL-243; RRID: CVCL_0004) or desired target cell line.
  **CAUTION** Cell lines should be regularly screened to ensure they are authentic and free of mycoplasma contamination.

### Reagents

#### Plasmids

- Super PiggyBac Transposase expression vector (System Bioscience, cat. no. PB210PA-1; or equivalent)
- PiggyBac-TRE-PEmax-T2A-mCherry-PGK-rtTA-P2A-Puro (Addgene, cat no. 239974)
- PiggyBac-ENGRAM backbone (Addgene, cat no. 239972)
- PiggyBac-synHEK3-Tape (Addgene, cat no. 239975)
- PiggyBac-5x-Typewriter-Tape (Addgene, cat no. 239983)

#### Cloning of DNA plasmids

- Nuclease-free water (Invitrogen, cat. no. AM9937)
- Q5^®^ High-Fidelity 2X Master Mix (NEB cat. no. M0492L)
- KAPA2G Robust 2X Hotstart mix (Kapa, cat. no. KK5702)
- SYBR Green (Thermo Fisher, cat. no. S7563)
- Agencourt AMPure XP (Beckman Coulter, cat. no. A63882)
- LB medium (e.g. Thermo Fisher, cat. no. 12795027)
- LB agar medium (e.g. Thermo Fisher, cat. no. 22700025)
- Ampicillin sodium salt (Fisher Scientific, cat. no. BP1760–25)
- Monarch Spin Plasmid Miniprep Kit (NEB, cat. no. T1110S)
- ZymoPure Midi kit (Zymo, cat. no. D4201)
- BsaI-HF-v2 (NEB, cat. no. R3733S)
- BsmBI-HF-v2 (NEB, cat. no. R0739S)
- BbsI-HF (NEB, cat. no. R3539S)
- 10× rCutSmart buffer (NEB, cat. no. B6004S)
- 10× NEBuffer^™^ r3.1 (NEB, cat. no. B6003S)
- NEBridge Ligase Master Mix (NEB, cat. no. M1100S)
- T4 DNA ligase (NEB, cat. no. M0202S)
- 10× T4 DNA ligase reaction buffer (NEB, cat. no. B0202S)
  **CRITICAL** Multiple freeze-thaw cycles will cause the ATP in the 10x T4 DNA ligase reaction buffer to degrade. Divide into aliquots of working volumes to minimize freeze-thaw cycles, and store as recommended by the manufacturer.

#### Mammalian cell culture

- DMEM (Thermo Fisher, cat. no. 119650192)
- RPMI 1640 (Thermo Fisher, cat. no. 11875093)
- FBS characterized (Cytiva, cat. no. SH30396.03)
- Penicillin-streptomycin (100× concentration) (Gibco, cat. no. 15140–122)
- Puromycin dihydrochloride (Gibco, cat. no. A1113803)
- Blasticidin S HCl (Thermo Fisher, cat. no. A1113903)
- Dulbecco’s PBS 1× (Gibco, cat. no. 14190–144)
- TrypLE^™^ Express Enzyme (Thermo Fisher, cat. no. 12604013)
- Lipofectamine 3000 transfection reagent kit (Thermo Fisher, cat. no. L3000001)
- Opti-MEM reduced serum medium (Thermo Fisher, cat. no. 31985062)
- CHIR-99021(Selleck, cat. no. S2924)
- Recombinant Human TNF-alpha Protein (R&D Systems, cat. no. 210-TA-005/CF)
- Doxycycline hyclate (Dox; Sigma, D9891)
- Proteinase K (Thermo Fisher, cat. no. EO0491)
- UltraPure 1M Tris-HCI, pH 8.0(Thermo Fisher, cat. no. 15568025)
- Ultrapure SDS 10% solution (Thermo Fisher, cat. no. 15553027)
- (Optional) Dneasy blood & tissue kit (Qiagen, cat. no. 69504)
- SF Cell Line 4D-Nucleofector X Kit S (Lonza, cat no. V4XC-2032)

#### Sequencing library preparation

- NanoDrop spectrophotometer (Thermo Fisher, cat. no. ND-8000-GL; or DNA concentration quantification tool alternatives)
- Agilent 4200 TapeStation (Agilent, cat. no. G2991BA; or DNA size quantification tool alternatives)
- Illumina NextSeq 1000/2000 system (Illumina, cat. no. 20038897; or alternatives)

#### General equipment

- Magnetic 96-well PCR plate (Beckman Coulter, cat. no. A32782 or similar)
- Filtered pipette tips, assorted (Fisher, cat. nos. 12-111-132, 76175-406, 12-111-002 and 12-111-000)
- Serological pipettes, assorted (Eppendorf, cat. nos. 30127714, 30127722, 30127730 and 30127749)
- Standard microcentrifuge tubes (Eppendorf, cat. no. 30108418)
- Standard PCR eight-strip tubes (USA Scientific, cat. no. 1402–4700)
- Standard cryogenic vials for liquid nitrogen storage (VWR, cat. no. 89089–764)
- CoolCell (Corning, cat. no. 432000)
- Vortex mixer (VWR, cat. no. 10153–838)
- Benchtop microcentrifuge (Eppendorf, cat. no. 5405000441)
- Countess automated cell counter (Thermo Fisher, cat. no. AMQAX2000)
- BD FACS Symphony S6 cell sorter (or alternative)
- Light microscope with RFP, CFP, YFP excitation laser and filter (Zeiss Axio Observer 3 or similar)
- 4D-Nucleofector^®^ System (Lonza, cat. No. AAF-1003)

### Reagent setup

#### DMEM (with 10% (vol/vol) heat-inactivated FBS)

Supplement DMEM with 10% (vol/vol) heat-inactivated FBS and 1% (vol/vol) penicillin–streptomycin. Store at 4 °C for up to 3 months.

#### RPMI 1640 (with 10% (vol/vol) heat-inactivated FBS)

Supplement DMEM with 10% (vol/vol) heat-inactivated FBS and 1% (vol/vol) penicillin–streptomycin. Store at 4 °C for up to 3 months.

#### Cell lysis buffer

To prepare 50 mL of cell lysis buffer, combine 500 μL of 1 M Tris-HCl (pH 8.0) and 250 μL of 10% SDS. This solution, *without* Proteinase K, can be stored at room temperature (25°C) for up to 6 months. Immediately before cell lysis, add Proteinase K to a final concentration of 40 μg/mL by adding a 1:500 (v/v) dilution of a Proteinase K stock solution (Thermal Fisher, ~20mg/ml). The final lysis buffer composition will be 10 mM Tris-HCl (pH 7.5), 0.05% SDS, and 40 μg/mL Proteinase K.

## Procedures

### Procedure 1: Generating monoclonal cell lines bearing the prime editor and synthetic DNA Tape

#### Timing: Approximately 30 days

This procedure establishes the essential groundwork for recording experiments, where we provide detailed instruction to engineer monoclonal cell lines with foundational recording machinery (prime editor and synthetic DNA Tape) for subsequent signal and CRE activity recording using ENGRAM.

#### Making UMI-labeled DNA Tapes (Optional)

##### Timing: 2 days

1. Perform PCR on PiggyBac-synHEK3-Tape plasmid with BsaI_15N_fwd primer and Tape_ampup_rev primer using Q5 polymerase.

**Table:** 

	Volume (uL)
Q5 DNA polymerase 2X master mix	25
10 μM BsaI_15N_fwd	2.5
10 μM Tape_ampup_rev	2.5
1 ng/μL template	1
Water	19
Sum	50
  With the following PCR conditions:

**Table:** 

Temperature	Duration	Number of cycles
98°C	30 seconds	1
98°C	10 seconds	15–20 cycles
60°C	15 seconds	
72°C	30 seconds	
72°C	1 minutes	1
4°C	Hold	1
2. Clean and concentrate the PCR product using AMPure XP beads by following the manufacturer’s instructions (or column-based DNA purification methods). Use the D1000 Tapestation to confirm the size and concentration. This product can be used for both PiggyBac-synHEK3-Tape and PiggyBac-5x-Typewriter-Tape.
3. Clone the 15N PCR product into PiggyBac-synTape plasmid backbone using Golden Gate Assembly with BsaI (Box 1).
  Troubleshooting
  Box 1. **One-pot Golden Gate Assembly (GGA) protocol.**

**Table:** 

Component	Amount
Plasmid	50 ng
Inserts	5–10ng (5:1 molar ratio)
NEBridge Ligase Master Mix (3X)	4 μL
BsaI-HFv2 (20 U/μL) or BsmBI-HFv2 (20 U/μL)	1 μL (20 units)
Nuclease-free H_2_O	to 12 μL

  **Critical step**: The reaction volume can be adjusted from 6 μL to 20 μL depending on the complexity and amount of plasmid in the reaction. Alternatively, the Ligase Master Mix can be substituted with 400 U of T4 DNA ligase and 1X T4 ligase buffer.
  Perform the Golden Gate Assembly (GGA) reaction using the following protocol:

**Table:** 

Restriction Enzyme(s)	Thermocycling Conditions
BsaI-HFv2, BbsI-HF	15 cycles of 37°C for 1 min, followed by 16°C for 1 min. 60°C for 5 minutes to deactivate ligase.
BsmBI-v2	15 cycles of 42°C for 1 min, followed by 16°C for 1 min. 60°C for 5 minutes to deactivate ligase.
  **Critical step**: consider using 30 cycles for thermocycling in complex libraries.
4. Transform 2 μL of the GGA product into 50 μL NEB 10-beta Electrocompetent *E. coli* competent cells by electroporation, following the manufacturer’s instructions.
  **Critical step**: The buffer in GGA might affect the electroporation. 2 μL GGA reaction with 50 2 μL competent cells are normally fine in our experience. If necessary, purify the plasmid from the GGA reaction using 0.5 X AMPure XP beads by following the manufacturer’s instructions.
5. Add 948 μL Stable Outgrowth Medium and recover the transformed cells by incubating them at 30°C for 30 minutes with 220 rpm shaking.
  **Critical step**: Bacteria with PiggyBac constructs should grow at 30 °C rather than 37 °C. Growth at 37 °C induces higher rates of plasmid recombination due to the repetitive sequences such as the inverted terminal repeats (ITRs). All bacterial cultures in this protocol are grown at 30°C.
6. Take 10 μL aliquot of the recovered cells and plate onto selective LB agar plates containing 100 μg/mL Ampicillin. Transfer the remaining outgrowth medium into a 50 mL culture tube containing LB medium supplemented with 100 μg/mL Ampicillin. Culture the cells at 30°C for approximately 18 hours.
  **Critical step:** The culture might need to be further diluted 100 times before plating onto the plate for counting.
7. Count the number of colony-forming units (CFUs). A library size exceeding 10,000 unique barcodes is generally recommended for sufficient complexity.
8. Purify the plasmid DNA using a Zymo Midi-Prep kit or an equivalent plasmid purification method, following the manufacturer’s instructions. Ensure the eluted DNA is of high quality and concentration for downstream applications.

#### Generating a stable cell line expressing PEmax and containing the synthetic DNA Tape using the PiggyBac transposon system

##### Timing: Approximately 7–10 days

- Seed 5 × 10^5^ cells into a 6-well plate a day before transfection.
- On the day of transfection, prepare a transfection mixture containing 1200 ng PB-PEmax, 800 ng PB-synTape, and 400 ng PB transposase expression plasmid, at a cargo : transposase molar ratio of 4:1.
  **Optional:** To generate cell lines with high copy numbers of DNA Tape, the molar ratio between the selection marker–containing PEmax plasmid and the selection-free DNA tape plasmid can be adjusted during transfection. Reducing the molar ratio of PEmax:synTape from 1:3 to 1:10 or 1:20 and select cells based on the Puromycin selection marker can result in lines with high (~60–100) copy numbers of DNA Tapes.
- Transfect the plasmid mixture into the target cells using Lipofectamine 3000 (or Lipofectamine 2000 for mESCs), following the manufacturer’s instructions.
  **Critical step:** Here we describe a protocol for adherent cell lines. This protocol can be adopted for non-adherent cell lines such as K562 by replacing Lipofectamine 3000 with Lonza SF Cell Line 4D-Nucleofector kits to maintain transfection efficiency.
- Change the medium the day after transfection and culture the transfected cells in their standard growth medium for an additional 48 hours to allow for sufficient expression of the transposase and integration of the cargo into the genome.
- Pass the transfected cells into a T25 tissue culture flask and initiate selection of successfully integrated cells by adding puromycin to the growth medium at a final concentration of 2 μg/mL.
  **Critical step:** Integrating large genetic payloads like PEmax using transposon systems can be relatively inefficient. Expect significant cell death (up to 90%) during the initial selection period. Regularly monitor the cells and replenish the selection medium every 2–3 days.
- Continue culturing the cells in puromycin-containing medium for up to 10 days, or until a population of stably resistant cells emerges.

#### Selection of single-cell-derived clones for robust and efficient recording, and sequencing the DNA Tape to create a whitelist of known DNA Tapes (Optional but highly recommended)

##### Timing: Approximately 14 days

**Figure 3.**
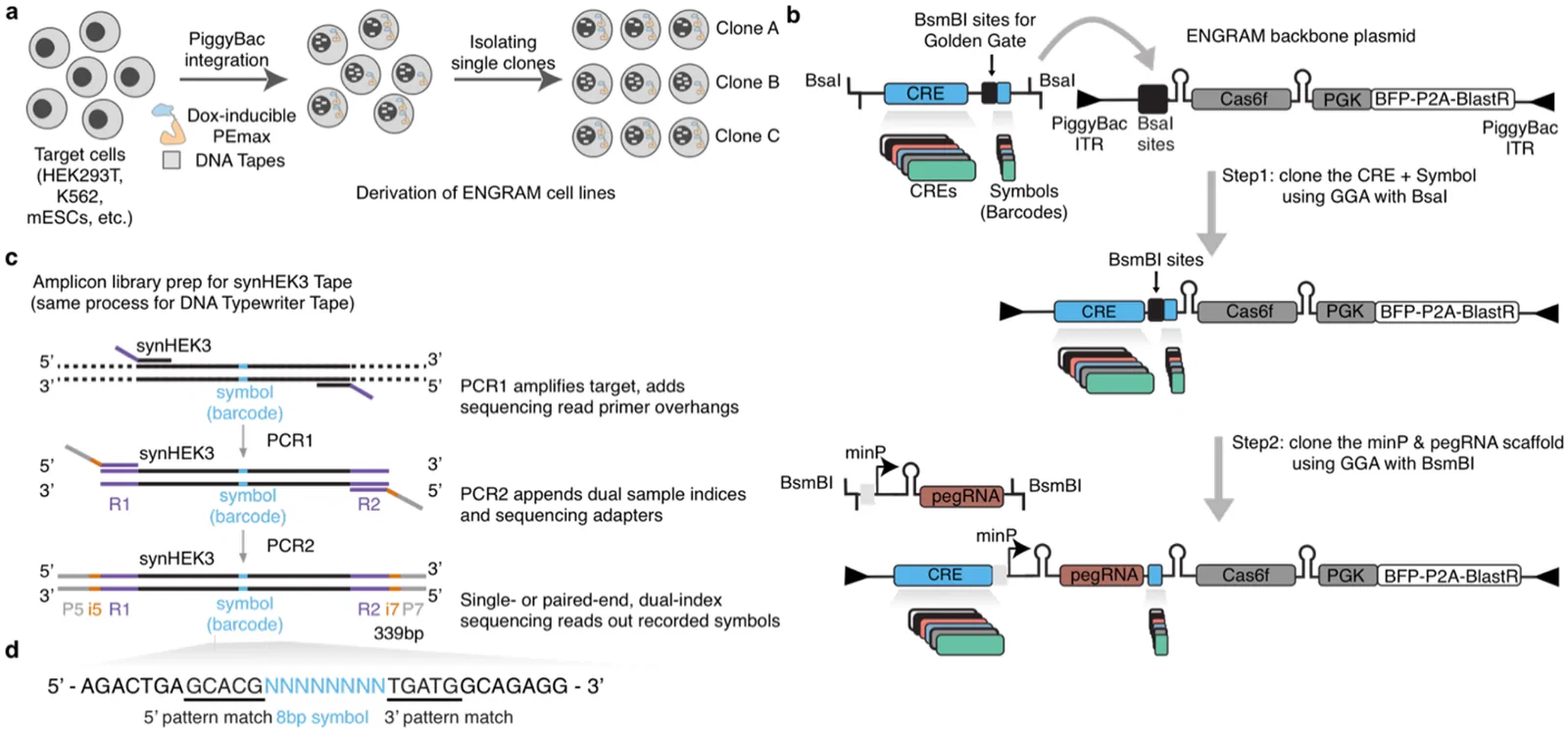
Overview of ENGRAM experimental procedures. **a**. Schematic of making ENGRAM cell lines. Dox-inducible PEmax and DNA Tapes are integrated into the genome via PiggyBac transposition. Deriving a monoclonal ENGRAM cell line bearing these components is recommended for robust and reliable molecular recording. **b**. Framework of two-step Golden Gate Assembly to clone ENGRAM pegRNAs with paired symbols (barcodes). In Step 1, a library of CRE-symbol pairs is cloned into the PiggyBac-ENGRAM backbone via Golden Gate Assembly with BsaI. In Step 2, an dsDNA molecule containing: (i) a minimal promoter (minP); and (ii) a pegRNA scaffold flanked on the 5’ end by a Cas6f hairpin, is cloned into the constructs derived from Step 1 via Golden Gate Assembly with BsmBI. **c**. Schematic of nested PCR amplicon sequencing strategy to recover recorded information from DNA Tape. The two step nested PCR amplicon library prep process is essentially the same for both kinds of DNA Tapes described in this protocol (synHEK3 and DNA Typewriter; synHEK3 visualized here). The first PCR amplifies the target and adds partial sequencing primer overhangs. The second PCR appends dual sample indices and sequencing adapters. Single- or paired-end, dual-index high-throughput sequencing is used to demultiplex samples (index reads i5 and i7) and recover recorded information (sequencing reads R1 and/or R2). **d**. Pattern matching functions are used to extract recorded information written as specific programmed symbols to the DNA Tape.

- Induce the expression of PEmax-T2A-mCherry by adding doxycycline to the cell culture medium at a final concentration of 200 ng/mL.
- Perform fluorescence-activated cell sorting (FACS) to isolate single cells into individual wells of a 96-well plate. Gate on cells exhibiting medium to high expression of PEmax-T2A-mCherry.
  **Optional:** Changing the gating of mCherry expression will alter the copy number of PEmax in the cells. If a FACS instrument is not available, we recommend isolating single-cell–derived clones by limiting dilution.
- Allow the sorted single cells to grow and expand in their respective wells for approximately one week, or until visible colonies form. Change the culture medium as needed to ensure proper cell growth and viability.
- Select 12 colonies and transfer them to a new plate for further expansion. Maintain proper labeling and tracking of each clone.
- To assess the recording efficiency of these clones, induce the expression of PEmax by adding doxycycline to the cell culture medium at a final concentration of 200 ng/mL. Let the cell express for 2 days.
- Transiently transfect each clone with a plasmid expressing a pegRNA targeting synTape (e.g. U6-HEK3-pegRNA or U6-DTT-pegRNA).
- After 48 hours, harvest the cells and prepare the library to analyze the recording efficiency (See Box 2).
  Box 2. **Retrieval of Recording Information from DNA Tape: Lysis and PCR**
  This section details the steps for lysing cells and extracting DNA for PCR amplification (Figure 3) of DNA Tapes.
  i. Determine the number of cells using a cell counter (e.g. using Countess automated cell counter). Accurate cell counting is crucial for standardizing the DNA extraction process.
  i. Lyse the cells at a concentration of 1 million cells per 200 μL of lysis buffer. Gently resuspend the cell pellet in the lysis buffer by pipetting up and down several times. Ensure the pellet is completely dispersed to facilitate efficient lysis.
  ii. Incubate the cell lysate at 55°C for 1h, followed by an 80 °C enzyme inactivation step for 30 min.
    - Cell lysis at high cell concentrations will initially result in a viscous solution. Once the digestion is complete, the solution should return to normal viscosity.
    - The described lysis buffer method is suitable for precious samples with low cell numbers, such as organoids. Please estimate the cell number and adjust the volume of lysis buffer accordingly.
    - Alternatively, genomic DNA (gDNA) can be purified using the DNeasy Blood & Tissue Kit (Qiagen) or equivalent, following the manufacturer’s instructions.
  iii. PCR-1: amplify up the synthetic Tape using Tape_ampup_fwd and Tape_ampup_rev, with 2x Kapa Robust DNA polymerase 2X master mix, with the condition below:

**Table:** 

	Volume (μL)
Kapa2G Robust HotStart ReadyMix	25
10 μM Tape_ampup_fwd	2
10 μM Tape_ampup_rev	2
Cell lysis from above	4 (equivalent 20,000 cells)
100X SYBR green	0.5
Water	32
Sum	50
    **Critical step:** remove qPCR samples when the amplification curve begins, typically between 18–22 cycles, depending on the integrated DNA Tape copy number of the selected clone. Once the monoclonal line is established and the required PCR cycle number is confirmed, standard PCR with a fixed number of cycles can replace qPCR. Over-amplification will introduce PCR bias and require deeper sequencing to recover information. We suggest having a lower number of cycles for the step 1 PCR.
    **Optional:** Unique Molecular Identifiers (UMIs) can be employed to improve the quantification of recorded information. However, as the DNA Tape is usually abundant (i.e. many copies per genome equivalent) and PCR amplification is targeting the same sequence, our experience to date suggests that PCR introduces minimal amplification bias, provided that one begins with sufficient genomic DNA. Because attaching UMIs to genomic DNA requires an additional step prior to PCR and complicates downstream analysis, we do not typically use UMI-based approaches when reading out ENGRAM from bulk genomic DNA. We note however that UMIs are a standard component of most single cell molecular profiling protocols, and as such are straightforward to take advantage of when ENGRAM records are being read out by scRNA-seq.

**Table:** 

Temperature	Duration	Number of cycles
95°C	60 seconds	1
95°C	10 seconds	18–22 cycles
65°C	15 seconds	
72°C	30 seconds	
72°C	1 minutes	1
4°C	Hold	1
  iv. Confirm precise amplification of amplicons using TapeStation or gel electrophoresis. The length of PCR-1 should be around 230 bp (Supplementary Table 1).
  v. PCR-2: adding sequencing adaptors P5_bc_Nextera and P7_bc_Truseq to PCR-1. Taking 1 μL of PCR-1 product to the following PCR mix.

**Table:** 

	Volume (μL)
Kapa2G Robust HotStart ReadyMix	12.5
10 μM P5_bc_Nextera	1
10 μM P7_bc_Truseq	1
PCR-1 product	1
Water	9.5
Sum	25
    With the following PCR conditions:

**Table:** 

Temperature	Duration	Number of cycles
95°C	60 seconds	1
95°C	10 seconds	5 cycles
65°C	15 seconds	
72°C	30 seconds	
72°C	1 minutes	1
4°C	Hold	1
    **Critical step**: Ensure PCR-1 is not overamplified. The overamplification of PCR-1 will lead to the incomplete adaptor attachment during PCR-2, resulting in two bands on a gel.
    Example primers are provided in Supplementary Table 1.
  vi. Pool samples with equal molar and clean up using AMPure XP beads by following the manufacturer’s instructions.
    **Critical step**: mixing samples after PCR-2 and purifying together relies on the same amount of input at PCR-1, which leads to similar concentration of PCR products in PCR-2.
  vii. Confirm precise amplification of amplicons using TapeStation or gel electrophoresis. The length of step 1 PCR should be around 330 bp. (Supplementary Table 1).
    **Critical step**: AMPure bead cleanup effectively removes primer dimers. However, if two bands are present, gel extraction is recommended to purify the band around 330 bp.
  viii. Quantify the PCR-2 product using TapeStation HS D1000 or Quibt. Dilute the sample to 2 nM and load it to the Illumina Nextseq 1000/2000 system (or similar high-throughput sequencing instruments), following the manufacturer’s instructions.
    - Typically, the P1 100 cycle kit should cover the full length of the synHEK3 Tape (using single-end sequencing),
    - P1 300 cycle kit should cover 5X-DTT Tape. While the recording efficiency of later Tapes is low, the P1 100 cycle kit with single-end sequencing should cover information stored in the first 3 Tapes as well.
  ix. Using the analysis script to calculate the recording efficiency of each clone. Estimate the copy number of synTapes in each clone by counting the number of unique intBCs.
    - For synHEK3, the inserted barcode is extracted using pattern matching, and its frequency is quantified.
    - For 5X-DTT, sequencing reads are first aligned to a reference sequence, and a custom Python script is used to extract information and calculate recording efficiency.

### Procedure 2: Cloning ENGRAM recorders targeting synHEK3 or Typewriter Tape

#### Timing: 4–5 days

This procedure provides detailed instructions to design, clone, and prepare CRE-specific ENGRAM recorders for signal recording (Procedure 3) and CRE activity recording (Procedure 4).

- Use the provided custom script to assign a unique barcode (BC) to the CRE of interest. Order the designed CRE-BC oligos from Twist Bioscience or similar services.
  **Critical step**: Recording efficiency has been measured for 5N and 8N barcode libraries. For better data interpretation, balanced recording efficiency across different barcodes is critical.
  **Critical step:** Long, homologous, intervening sequences between a CRE and its paired BC can also lead to barcode swapping during amplification^62^ or pooled viral packaging^63,64^. Barcode swapping can introduce inaccuracies in recording and compress signal-to-noise. CRE-BC distance should be minimised for ENGRAM libraries (e.g. as in the 5’-ENGRAM design and PiggyBac cloning scheme described with which we observe little to no swapping). For this reason, we recommend **not** adapting this protocol for Gibson assembly, as we have seen that Gibson assembly increases the rate of CRE-BC swapping. To clone signal-specific CREs, we recommend using specific primer pairs to amplify the oligo from the pool.
  Troubleshooting
- Clone ENGRAM recorders with a 2-step nested Golden Gate Assembly
  - **Generation of individual signal-specific ENGRAM recorders**
    - Amplify signal-specific CREs with unique PCR primer pairs, using the following mix and qPCR conditions.

**Table:** 

	Volume (uL)
Q5 DNA polymerase 2X master mix	25
10 uM CRE_fwd	2.5
10 uM CRE_rev	2.5
1 ng/μL template	1
100X SYBR green	0.5
Water	18
Sum	50

**Table:** 

Temperature	Duration	Number of cycles
98°C	30 seconds	1
98°C	10 seconds	10–15 cycles
60°C	15 seconds	
72°C	45 seconds	
72°C	5 minutes	1
4°C	Hold	1
      **Critical step** - Typically, we recommend performing qPCR so that the product can be monitored in order to minimize the number of PCR cycles needed. We recommend 10–15 cycles, but you can go to 20 cycles of PCR if necessary.
    - Clean and concentrate the PCR product using AMPure XP beads by following the manufacturer’s instructions (or column-based DNA purification methods). Use the D1000 Tapestation to confirm the size and concentration.
    - Clone the CRE-BC PCR product into PiggyBac-ENGRAM plasmid backbone using Golden Gate Assembly with BsaI (See Box 1).
    - Transform 1μLof GGA product into 10μL NEB C3040 competent cells by following the manufacturer’s protocol.
    - Plate the transformation reaction onto an LB agar plate containing 100 μg/mL ampicillin and incubate overnight at 30 °C.
    - Pick colonies for whole plasmid sequencing using Plasmidsaurus’ ZeroPrep service by following their instructions.
    - Expand the sequence verified colonies for mini prep.
    - Clone the minP-pegRNA DNA fragment into the plasmid prepared from last step using GGA with BsmBI (See Box 1).
    - Transform 1μLof GGA product into 10μL NEB C3040 competent cells by following the manufacturer’s protocol.
    - Plate the transformation reaction onto an LB agar plate containing 100 μg/mL ampicillin and incubate overnight at 30 °C.
    - Pick colonies for whole plasmid sequencing using Plasmidsaurus’ ZeroPrep service (or an alternative plasmid sequencing service) by following their instructions.
  - **Generation of a library of CRE-ENGRAM recorders**
    - Perform the Step 1 BsaI Golden Gate Assembly as described in step 23 (A, i-vi).
    - Transform 2 μL of the GGA product into 50 μL NEB 10-beta Electrocompetent *E. coli* competent cells by electroporation, following the manufacturer’s instructions. If you have a large library, repeat electroporation multiple times to achieve enough coverage.
      **Critical step**: The high salt buffer in GGA might affect the electroporation. If necessary, purify the plasmid from the GGA reaction using 0.5 X AMPure XP beads by following the manufacturer’s instructions.
    - Add 948 μL Stable Outgrowth Medium and recover the transformed cells by incubating them at 30°C for 30 minutes with 220 rpm shaking.
    - Take 10 μL aliquot of the recovered cells and plate onto selective LB agar plates containing 100 μg/mL Ampicillin. Transfer the remaining outgrowth medium into a 50 mL culture tube containing LB medium supplemented with 100 μg/mL Ampicillin. Culture the cells at 30°C for approximately 18 hours.
      - The culture might need to be further diluted 100 times before plating it onto the plate for counting.
    - Count the number of colony-forming units (CFUs). A CFU more than 1,000 x the number of elements in the library is generally recommended for sufficient complexity.
    - Purify the plasmid DNA using a Zymo Midi-Prep kit or an equivalent plasmid purification method, following the manufacturer’s instructions. Ensure the eluted DNA is of high quality and concentration for downstream applications.
      - **Critical step**: it is recommended to quantify the complexity of the library at each step. We recommend performing PCR and Next-generation sequencing to estimate the coverage of the CREs and estimate the ratio of barcode swapping. The only source of barcode swapping so far is the chimeric PCR product during the oligo pool amplification, which is normally ~1 %. For sequencing library preparation, please refer to Box 2 iv–ix.
    - Clone the minP-pegRNA DNA fragment into the plasmid prepared from the last step using GGA with BsmBI (See Box 1).
    - Repeat steps (ii-iv), transform 2 μL of the GGA product into 50 μL NEB 10-beta Electrocompetent *E. coli* competent cells by electroporation.
      - **Critical step**: Purify the plasmid from the GGA reaction using 0.5 X AMPure XP beads for better electroporation.
      - If you have a large library, repeat electroporation multiple times to achieve enough coverage.
    - Count the number of colony-forming units (CFUs).
    - Purify the plasmid DNA using a Zymo Midi-Prep kit or an equivalent plasmid purification method, following the manufacturer’s instructions. Ensure the eluted DNA is of high quality and concentration for downstream applications.
    - Verify the plasmid pool by PCR and sequencing the library with two step PCR.

### Procedure 3: Recording signaling pathway activity and order with ENGRAM

#### Timing: 15–18 days

To illustrate signal recording and intensity measurement, we use the Wnt and NFκB signaling pathways as examples. This protocol can be adapted to study other signaling pathways by utilizing appropriate signal-responsive CREs. The protocol is compatible with both synHEK3 and Typewriter Tapes. synHEK3 Tape is recommended for quantifying signal intensity due to its efficiency, whereas Typewriter Tape should be used for recording the temporal order of signals. In this protocol description, the specific recorders and their corresponding synHEK3 or Typewriter Tape are not explicitly mentioned; users should ensure they select the appropriate Tapes and recorders for their intended application.

#### Making cell lines with signal-specific ENGRAM

##### Timing: 7–10 days

- Seed 5 × 10^5^ monoclonal cells derived from steps 15–21 into a 6-well plate a day before transfection.
- On the day of transfection, prepare a transfection mixture containing 2000 ng signal-specific PB-ENGRAM-recorder and 400 ng PB transposase expression plasmid at a molar ratio of 5:1.
- Transfect the plasmid mixture using Lipofectamine 3000 following the manufacturer’s instructions.
  - Optional: Transiently transfecting the ENGRAM recorder may result in low background recording. While this background is minimal in TRE-PEmax cell lines, it can be further reduced by co-transfecting 1000 ng of a U6 promoter-driven non-targeting pegRNA (NTC – not provided; any gRNA or pegRNA should be effective).
- Change the medium the day after transfection and culture the transfected cells in their standard growth medium for an additional 48 hours to allow for sufficient expression of the transposase and integration of the cargo into the genome.
- Pass the transfected cells into a T25 tissue culture flask and initiate selection of successfully integrated cells by adding blasticin to the growth medium at a final concentration of 10 μg/mL.
  **Critical step:** PB-ENGRAM cargo is much smaller than PEmax. Most of the cells should survive and contain a few copies of the recorder. Regularly monitor the cells and change the selection medium every 2–3 days.
- Continue culturing the cells in blasticin-containing medium for up to 10 days, or until a population of stably resistant cells emerges.

#### Recording the intensity of signals

##### Timing: 2 days

- Prepare signal ligand medium: For Wnt and NFκB signaling pathways, create 12 serial dilutions of CHIR-99021 (0.25 μM to 16 μM) and human TNFα (starting from 64 ng/mL), respectively, using a 2X stock. For CHIR-99021, include additional concentrations between 2 μM and 4 μM to cover its sensitive range.
- Count cells and dilute to 4 × 10^5 cells/mL. Seed 250 μL of this suspension into each well of a 48-well plate.
- Dispense 250 μL of the 2X ligand medium serial dilutions into designated wells to achieve the intended final ligand concentrations. Ensure each condition is tested in triplicate for robust recording measurement.
- Culture the cells in ligand containing medium for 48 hours.
- Harvest the cell and retrieve the recorded information by following the steps in Box 2.
  **Critical step**: Typically, a confluent well of a 48-well plate contains approximately 1 million cells. The protocol can be slightly modified as follows: Aspirate the medium, add 200 μL of freshly prepared lysis buffer (10 mM Tris-HCl, pH 8; 0.05% SDS; 40 μg/mL proteinase), and incubate at 37°C for 30 minutes. Transfer the lysate to a PCR tube and follow the protocol described in Box 2. For signal recording, 1 PCR reaction is sufficient to recover the information.
  Troubleshooting

#### Recording the order of signals

##### Timing: 4–6 days

- Prepare 2x signal ligand medium for each signal. For example, 6 μM CHIR-99021 for Wnt signaling pathway (signal A) and 20 ng/mL human TNFα for NFκB signaling pathway(signal B).
- Count cells and dilute to 4 × 10^5 cells/mL. Seed 250 μL of this suspension into 6 wells of a 48-well plate, representing two orders of signals (A→ B, and B→ A) and three replicates each.
- Dispense 250 μL of the 2X ligand medium serial dilutions into designated wells to achieve the intended final ligand concentrations.
- Culture the cells for 48 hours for sufficient recording.
- Passage the cells into new wells at a 1:4 ratio. Culture these cells in medium containing the alternative ligand for an additional 48 hours.
- Harvest the cell and retrieve the recorded information by following the steps in Box 2.

### Procedure 4: Multiplex recording of enhancer activities with ENGRAM

#### Timing: 15–18 days

To illustrate multiplex enhancer activity measurement, we used validated enhancers in K562 cells and assigned unique barcodes (symbols) to them.

#### Making cell lines with the CRE-ENGRAM library

##### Timing: 2 days

We have tested multiplex CRE recording in K562 cells. This procedure requires K562 cells with TRE-PEmax and synHEK3 DNA Tape.

- Culture K562 cells in RPMI 1640 medium for 3 days prior to transfection. Confirm sufficient cell quantity for the procedure.
- To determine the required number of cells for transfection, multiply the CRE library size by 1,000. For instance, if your CRE library contains 1,000 CREs, aim to transfect 1 million cells.
- On the day of transfection, prepare a transfection mixture containing 2000 ng signal-specific PB-ENGRAM-recorder and 400 ng PB transposase expression plasmid at a molar ratio of 5:1 and transfect into K562 cells with Lonza SF Cell Line 4D-Nucleofector kit by following manufacturer’s instructions.
  **Critical step:** This is an example of transfecting 1 million cells. Please scale the amounts of plasmids and reagents for transfection according to the calculated cell number from the previous step. A single cuvette can transfect upto 4 million cells. Repeat transfection three times as replicates.
- Culture the transfected cells in the standard growth medium for an additional 48 hours to allow for sufficient expression of the transposase and integration of the cargo into the genome.
- Pass the transfected cells into a T25 tissue culture flask and initiate selection of successfully integrated cells by adding blasticin to the growth medium at a final concentration of 10 μg/mL.
- Continue culturing the cells in blasticidin-containing medium for up to 10 days, or until a population of stably resistant cells emerges.

#### Recording CRE activities in cells

##### Timing: 2 days

- Induce recording by adding doxycycline to the cell medium at a final concentration of 200 ng/mL. Culture the cells for 48 hours to allow for sufficient recording of CRE activities. The optimal duration may vary depending on the cell type, CRE, and experimental goals.
- Concurrently, determine the necessary cell number to ensure adequate retrieval of recorded information. A general guideline is to aim for approximately 1,000 Tapes per CRE to achieve robust and statistically significant results. Consider the number of unique CREs in the library and the estimated number of DNA Tape copies per cell (determined at step 21 in each monoclonal line, e.g. 10–20 Tapes/cell). For instance, with 300 CREs and an estimated 10 Tapes per cell, performing 1–2 PCR reactions, which is equivalent to 20,000–40,000 cells, should ensure efficient information retrieval.
  **Critical step:** Determining the optimal number of reads needed for accurate measurement of CRE activity depends on several factors, including the library size and the proportion of active CREs it contains. As a general guideline, we recommend using ~1,000 tapes per CRE and 10,000–20,000 reads per CRE. For a library containing 10,000 CREs, approximately 100 million reads (e.g., from a single P1 kit) should provide reliable activity measurements. If the proportion of active CREs is expected to be low, the total number of reads can be reduced to around 10 million.
- Retrieve the recorded information by following the steps in Box 2. Perform PCR-1 with the number calculated in step 34, with the number of cycles obtained earlier for the clone you used (See Box 2).

## Anticipated results

Data from an ENGRAM recording experiment are counts of programmed symbols (insertional barcodes) extracted from reads of the recording DNA Tape (here sample demultiplexed high-throughput sequencing reads stored as FASTQ files). When combined with DNA Typewriter, the location of a given symbol within the sequential DNA Tape is an additional layer of information. From these counts of symbols and their sequential patterns in DNA Tape, several metrics can be quantified:

- The presence or absence of CRE/signal activity in a given sample during the recording period
- The fold induction, or signal-to-noise ratio between stimulated and unstimulated groups for each CRE/signal recorder (e.g. Figure 2b)
- The relative timing or order of each CRE/signal activity (inferred from bigram frequencies of given symbols from sequential DNA Typewriter records, e.g. Figure 2c)
- The relative strength of CREs/signals in a pool (e.g. Figure 2d–e)

When combined with single cell profiling methods, a cell barcode (CBC) can further be appended to sequencing reads of the DNA Tape, allowing the records to be related/combined with single cell profiles (e.g. single cell transcriptomes). This allows one to quantify the activity of given CRE/signals in a precisely defined transcriptomic cell type, or to reconstruct the history of cellular events (e.g. CRE/signalling activity) that produced a given cell type or state.

Key quality control metrics in an ENGRAM experiment include correlations in CRE activity (symbol counts and edit scores) between biological replicates, correlations in insertion counts between independent barcodes associated with the same CRE/ENGRAM recorder, overall prime editing efficiency (which facilitates information recovery), and signal-to-noise ratios between stimulated and unstimulated conditions. When executing or analyzing a recording experiment, a common diversion can be to unduly focus on increased signal (editing efficiency) rather than increased signal-to-noise (large fold-change between induced and control), the latter of which is considerably more valuable in many ENGRAM recording tasks (e.g. inferring if a CRE was ever active in a cell’s past). A successful experiment will allow the user to determine which CREs/signals are active in which samples or cells, as well as their relative intensity and/or orders with high reproducibility between biological replicates. We typically observe editing efficiencies of 20–40% with well characterized CREs in monoclonal PEmax cell lines (though editing efficiencies for different CREs can vary widely depending on their activity) and Pearson correlations >0.9 across replicates.

ENGRAM represents a new frontier where in theory any event that can be coupled to Pol-2 mediated transcription can be written permanently in DNA. When combined with DNA Typewriter, additional cellular events (lineage, time, protein-protein interactions, etc.) can be continuously and sequentially recorded to a universal DNA Tape. The combinatorial, single cell resolution recordings that this paradigm affords lends itself rapid adaptation to diverse signals of interest.

## Supplementary Material

ENGRAM_Nptrocol_SupplementaryTable1

## Figures and Tables

**Table 1.** Troubleshooting Table

Step	Problem	Possible reason	Solution
**Procedure 1**			
Step 3	Low barcoded DNA Tape cloning efficiency	Background with the backbone plasmid is high	Increase the number of Golden Gate assembly cycles to 50 cycles of 37°C for 1 min, followed by 16°C for 1 min. This applies to any step of this protocol in which Golden Gate assembly is used. Alternatively, pre-cut the backbone plasmid with BsaI followed by gel purification. This ensures no background plasmid is carried over.
**Procedure 2**			
Step 22	Swapping between CREs and barcodes	Too many PCR cycles were used to amplify the CRE-BC pars	Use fewer PCR cycles to amplify CRE-BC pairs. Alternatively, when ordering CRE-BC pairs as part of a pool with other elements, make the CRE-BC sequences a high fraction of the pool to reduce cycles required for dial-out PCR.
**Procedure 3**			
34	Low editing efficiency	Low copy number of ENGRAM recorders, TAPEs, or PEmax in the cells	Select cells with a higher number of ENGRAM recorders by performing FACS to isolate high fluorescence cell populations. If a drug resistance marker is on the ENGRAM plasmid, use a high dose of drug to select for cells with high ENGRAM copy number.
	Low editing efficiency	Editing time-window was too short	Increase the amount of time cells have to record ENGRAM events by waiting several more days to harvest, by changing the media to include fresh doxycycline every other day, and by adding fresh ligand as appropriate.
**Procedure 4**			
Step 49	Low editing efficiency	Editing time-window was too short	The majority of the CRE selected might be low activity or not active. Increasing the recording time helps to record these low activity CREs.

## Data Availability

Related scripts are available on GitHub (https://github.com/shendurelab/ENGRAM/tree/main/Nprotocol_scripts). The optimized ENGRAM plasmids have been deposited to Addgene with the following names and IDs: P021-mPB-ENGRAM-All-in-one-PGK-BFP-P2A-Blast (239972), P022-mPB-TRE-PEmax-T2A-mCherry-PGK-rttA-Puro (239974), P023-mPB-synHEK3-BsaI (239975), and P024-mPB-5xTAPE-BsaI (239983). Related raw data can be found in association with the supporting primary research articles.
