# Supplementary figures and images for: Multichannel Genomic Recording of Biological Information with ENGRAM
Source: Nat Protoc. Author manuscript; Available in PMC 2026 May 7. (PMC13150845; doi:10.1038/s41596-025-01322-w)

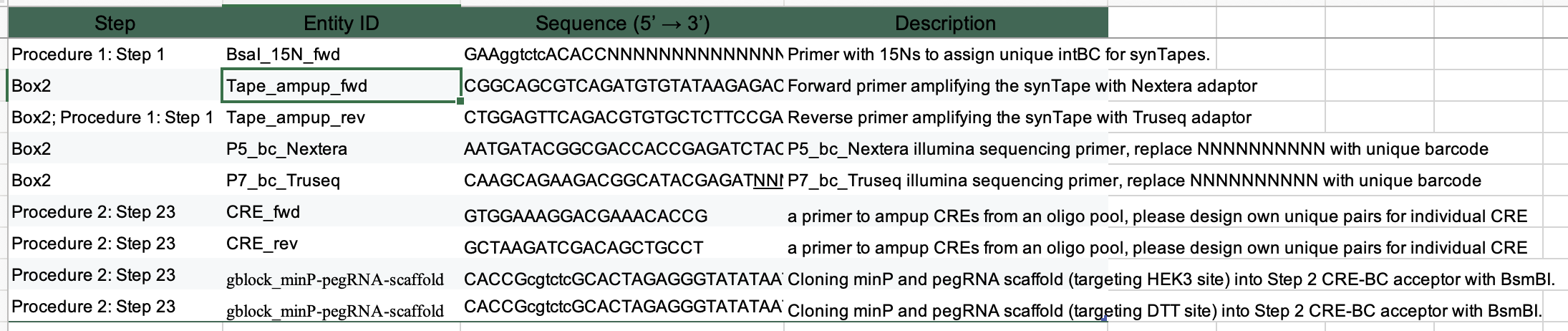

Supplement: ENGRAM_Nptrocol_SupplementaryTable1 [file NIHMS2167518-supplement-ENGRAM_Nptrocol_SupplementaryTable1.docx]
